# Current State of Knowledge of the Anticancer Properties of Polyphenolic Compounds from Garlic (*Allium sativum* L.)

**DOI:** 10.3390/molecules31050801

**Published:** 2026-02-27

**Authors:** Urszula Binduga, Konrad A. Szychowski

**Affiliations:** 1 Department of Civilization Diseases and Regenerative Medicine, Medical College, University of Information Technology and Management in Rzeszow, st. Sucharskiego 2, 35-225 Rzeszów, Poland; 2 Department of Biotechnology and Cell Biology, Medical College, University of Information Technology and Management in Rzeszow, st. Sucharskiego 2, 35-225 Rzeszów, Poland; kszychowski@wsiz.edu.pl

**Keywords:** garlic, polyphenols, anticancer, PPARs, ROS, Nrf2

## Abstract

Garlic (*Allium sativum* L.) belongs to the *Allium* genus and is one of the main bulbous plants consumed fresh, powdered, or cooked. Numerous studies have shown that garlic exhibits antihyperlipidaemic, antioxidant, anti-inflammatory, cardiovascular disease preventive, antihypertensive, antibacterial, antiviral, antifungal, antiparasitic, antidiabetic, anticarcinogenic, hepatoprotective, immunomodulatory, and hypoglycaemic effects. Moreover, studies on polyphenols detected in garlic reveal strong anticancer properties in various cell lines. The aim of this review is to summarise the current state of knowledge regarding the anticancer properties and shared molecular mechanisms of action of garlic-derived polyphenolic compounds. Our analysis demonstrates that the polyphenol content in garlic is highly variable and depends on numerous factors, including the part of the plant, processing methods, place of cultivation, and other conditions. Additionally, garlic contains polyphenols that exhibit anticancer activity in preclinical models, the properties of which have been demonstrated in in vitro studies. The anticancer mechanism of action varies depending on the type of polyphenol. Several polyphenols from garlic such as e.g., catechin, quercetin, and kaempferol activate peroxisome proliferator-activated receptors, which appear to contribute to at least part of garlic’s anticancer activity. The primary mechanism of garlic’s anticancer properties relies on reactive oxygen species-dependent toxicity and/or apoptosis, and Nrf2 is also implicated in the mechanism of action of garlic polyphenols. Our review provides evidence that under in vitro conditions, polyphenols present in garlic may exhibit anticancer properties. Garlic is not only a valuable culinary ingredient but also a natural medicine. Regular consumption in moderate amounts may offer numerous health benefits.

## 1. Introduction

Garlic (*Allium sativum* L.) belongs to the *Allium* genus and is one of the primary bulbous plants consumed fresh, powdered, or cooked. It is also traditionally used as a medicinal plant worldwide [1]. Fresh garlic cloves contain approximately 63% water, 28% carbohydrates, 7% protein, 0.2% fat, 0.8% fibre, and a significant amount of sulphur compounds. The composition and stability of these bioactive constituents can vary depending on processing and drying conditions [2,3]. These compounds contribute to the characteristic pungent taste and odour of garlic [4]. To date, it is widely accepted that garlic is rich in various biologically active substances [5]. Its diverse chemical composition provides a wide range of biological effects. Numerous studies indicate that garlic extracts or the consumption of raw cloves exhibit antihyperlipidaemic [6], antioxidant [7], anti-inflammatory [8], cardiovascular [9], antihypertensive [10], antibacterial [11], antiviral [12], antifungal [13], antiparasitic [14], antidiabetic [15], anticarcinogenic [16], hepatoprotective [17], immunomodulatory [18], and hypoglycaemic [19] effects.

Garlic’s biologically active compounds can be broadly classified into two groups: sulphur-containing and sulphur-free compounds [20]. According to recent reviews, both classes of compounds are strongly influenced by thermal and drying treatments, which can enhance or degrade allicin and phenolic content [2]. The first group includes alliin, γ-glutamylcysteine, and their derivatives such as allicin, diallyl sulphide (DAS), diallyl disulphide (DADS), diallyl trisulphide (DATS), ajoene, S-allylcysteine (SAC), and S-allylmercaptocysteine (SAMC) [20]. Garlic contains the highest concentration of sulphur compounds among all *Allium* species. Therefore, this class of compounds is widely considered responsible for its health-promoting properties [21]. However, the second group—sulphur-free compounds—also includes important bioactives, particularly polyphenols [22]. Despite being often underestimated, these compounds exhibit health-promoting properties comparable to, or even stronger than, those of sulphur-containing molecules [23]. Moreover, some polyphenol groups are present in higher amounts in aged garlic extract or black garlic (BG) compared to raw garlic [22,24]. Similar findings were reported by Thakur et al. (2025), who demonstrated that pre-treatment and drying technologies markedly influence the retention and transformation of phenolic compounds in garlic powder [3].

In 2022, an estimated 20 million new cancer cases and 9.7 million cancer-related deaths were reported worldwide [25]. As a result, the search for new therapeutic options and, more importantly, effective cancer prevention strategies is a major focus of the scientific community. Garlic, as a functional food, holds promise in this context. However, current data on the role and significance of garlic-derived polyphenolic compounds in cancer prevention remain fragmented and scattered. The aim of this manuscript is to summarise the current state of knowledge regarding the anticancer properties and molecular mechanisms of action of garlic-derived polyphenols.

Although the literature includes broad overviews of garlic’s health benefits, few focus specifically on the polyphenol-related anticancer mechanisms. For instance, Ahmed and Wang (2021) reviewed the bioactive compounds and health benefits of black garlic [26]. While they discussed its anticancer activity, they mostly catalogued general effects—such as antioxidant or anti-inflammatory actions—without exploring the molecular mechanisms of individual polyphenols. Similarly, Farhat et al. (2021) described various garlic preparations and their anticancer benefits, focusing primarily on organosulphur compounds and general antioxidant activity [27]. A recent review by Talib et al. (2024) also concentrated on garlic’s anticancer properties, mainly in the context of organosulphur constituents like allicin and ajoene [28]. They mentioned flavonoids and phenolics only briefly [28]. In contrast, the present manuscript offers a novel contribution by focusing on garlic-derived polyphenols (e.g., quercetin, caffeic acid, ferulic acid) and their specific anticancer mechanisms. This manuscript highlights how these polyphenols modulate key molecular targets—such as the phosphoinositide 3-kinase/protein kinase B (PI3K/Akt) survival pathway, the nuclear factor erythroid 2–related factor 2 (Nrf2) oxidative stress response, and transcription factors like nuclear factor kappa-light-chain-enhancer of activated B cells (NF-κB) and peroxisome proliferator-activated receptor gamma (PPARγ)—to induce apoptosis, inhibit tumour proliferation, and suppress metastasis. This mechanistic depth and focused analysis of polyphenolic compounds distinguish our work from prior garlic reviews. By doing so, we aim to fill a critical gap in understanding how garlic’s polyphenols exert anticancer effects at the molecular level.

## 2. Types of Polyphenolic Compounds in Garlic

Polyphenols are naturally occurring compounds primarily found in fruits, vegetables, and various plant-derived materials. To date, more than 8000 polyphenolic compounds have been identified in plants [29]. Typically, plants synthesise polyphenols in response to stress. These compounds help protect young plants from pests, ultraviolet radiation, and pathogens [30]. Polyphenols also influence key sensory and stability-related properties of food, including taste, colour, oxidative stability, and aroma. The polyphenol content in garlic is modulated by numerous factors. In addition to environmental stressors, these include the garlic variety, soil type, country of origin, sunlight exposure, rainfall, degree of plant maturity, harvest time, processing methods, and storage conditions [1,31,32].

Polyphenols can be classified into several groups based on the number of phenol rings and the structural linkages between them [22,33]. These groups include flavonoids (e.g., quercetin, catechins found in green tea) [34], phenolic acids (e.g., chlorogenic acid from coffee) [35], stilbenes (e.g., resveratrol from red wine) [36], and lignans (e.g., from linseed) [37].

Lignans are diphenol derivatives containing a dibenzylbutane skeleton. They exhibit characteristics similar to those of phytoestrogens [38]. It has been suggested that lignans may confer several health benefits, including antioxidant, antitumour, oestrogenic and anti-oestrogenic effects, as well as cardiovascular protection [39]. In the Mediterranean diet, lignan sources include garlic, onion, leafy and non-leafy vegetables, cereals, and seasonal fruits such as citrus. Each of these contributes differently—ranging from 11% to 70%—to the total polyphenol intake [40].

Stilbenes are composed of a benzoin ring structure with 15 carbon atoms and a phenylpropane unit. Their biological activity is determined by diverse structural modifications, which also overlap with those seen in flavonoids [41]. Although present in food only in small quantities, the best-known stilbene is resveratrol, found in red wine. Notably, studies on garlic from Nigeria have also identified resveratrol as the dominant stilbene in this variety [42].

Phenolic acids are generally classified into two types: benzoic acid derivatives and cinnamic acid derivatives. These compounds feature a benzoic acid ring with carboxyl and hydroxyl groups. Among them, hydroxycinnamic acids are more prevalent and include p-coumaric acid, caffeic acid, sinapic acid, and ferulic acid. In contrast, hydroxybenzoic acids include gallic acid, vanillic acid, p-hydroxybenzoic acid, and syringic acid [43]. Phenolic acids demonstrate strong antioxidant activity through multiple mechanisms. These include reducing activity (via electron or hydrogen donation), free radical scavenging, and interruption of radical chain reactions. Additionally, they act as oxidase inhibitors and metal ion chelators [44]. The antioxidant nature of phenolic acids significantly contributes to their recognized health-promoting effects.

Flavonoids are the largest and most extensively studied group of polyphenols. They are further classified into subgroups such as flavonols, flavones, catechins, proanthocyanidins, anthocyanidins, and isoflavonoids, as summarised in Table 1.

Many flavonoids and related compounds are known for their strong antioxidant properties [56]. They have been widely studied as potential bioactive ingredients for incorporation into functional foods. The primary activity of polyphenols is their ability to scavenge free radicals. This includes their capacity to chelate reactive metals and stabilise radicals [57]. However, under certain conditions, polyphenols may undergo oxidation. Factors contributing to peroxidation include alkaline pH, oxygen exposure, and high concentrations of specific metals [57]. The compound’s structure plays a key role in its radical scavenging activity, with critical features including the number of hydroxyl (-OH) groups and the degree of methylation [57]. The structure–activity relationships (SAR) of garlic-derived polyphenols are complex and cannot be fully generalised due to the structural diversity of these compounds. However, several consistent SAR patterns have been identified in the literature. In general, the anticancer and antioxidant activity of polyphenols strongly correlates with the number and position of hydroxyl (-OH) groups and the presence of conjugated π-electron systems. Hydroxycinnamic acids containing conjugated double bonds, such as caffeic acid and ferulic acid, typically exhibit greater biological activity than their non-conjugated analogues due to enhanced electron delocalisation and a radical stabilisation capacity [57,58,59]. Similarly, among flavonols, quercetin generally exhibits stronger antiproliferative and pro-apoptotic effects than kaempferol, which is attributed to the additional hydroxyl group at position 3′ of the B-ring. This structural feature enhances redox activity, increases ROS generation in cancer cells, and improves interaction with intracellular molecular targets [57,60,61]. Consistent with this observation, flavonols with a higher number of hydroxyl groups, such as quercetin and myricetin, typically demonstrate lower half maximal inhibitory concentration (IC_50_) values and greater cytotoxic potency compared with structurally related compounds containing fewer hydroxyl substituents [57,60,62,63]. These findings indicate that the number and arrangement of hydroxyl groups, as well as conjugation within the phenolic structure, are key determinants of anticancer activity. However, it should be emphasised that bioavailability, metabolic stability, and cellular uptake also play critical roles and may significantly influence biological activity independently of chemical structure.

Plants from the Allium genus, including garlic, are recognised for containing significant amounts of polyphenols [64]. According to data reported in the literature, raw garlic bulbs contain the highest levels of β-resorcylic acid, pyrogallol, p-hydroxybenzoic acid, and syringic acid (Table 2). Unfortunately, these compounds have not yet been studied in BG. A literature review shows that apigenin, kaempferol, and naringenin have not been detected in BG. Moreover, compounds such as caffeic acid, catechin, chlorogenic acid, epicatechin, gallic acid, and quercitrin are present in BG but in lower quantities than in raw garlic. BG is produced by aging whole garlic bulbs or separated cloves under controlled humidity (80–90%) and temperatures ranging from 60 °C to 90 °C, typically for 15 to 90 days [65]. In recent years, BG products have become increasingly popular in the Korean market as health-oriented foods, largely due to growing public awareness of garlic’s health benefits [22]. However, many health-promoting compounds may degrade during the aging process. This instability may account for the absence or reduced concentration of certain substances in BG compared to raw garlic. On the other hand, the concentrations of epigallocatechin gallate, m-coumaric acid, morin, o-coumaric acid, p-coumaric acid, quercetin, resveratrol, and vanillic acid are higher in BG. This increase is likely due to technological processing. Previous studies have shown that the BG production process increases total polyphenol and flavonoid content, while selectively enhancing certain compound classes [66]. A similar trend has been observed in other foods. For instance, during the aging of tangerine peel, flavonol levels tend to decrease, while isoflavones and chalcones increase. Dihydroflavones and flavanones decrease during the same process [67]. These findings support the hypothesis that technological processing significantly influences the polyphenol profile in BG.

Literature data also indicate that dried garlic contains lower levels of several polyphenols, such as catechin, kaempferol, protocatechuic acid, and rutin, compared to raw garlic. The exception is quercetin, which is found in higher concentrations in dried garlic. As discussed above, thermosensitive polyphenols can degrade during garlic processing. However, total polyphenol content generally increases during drying [68]. In onions, it has also been reported that quercetin levels rise in dried onion skins [69]. This observation is consistent with the current understanding from the literature on how technological processing influences polyphenol composition.

A comparison between garlic leaves and cloves showed that leaves contain significantly higher levels of apigenin, catechin, chlorogenic acid, ferulic acid, hesperidin, luteolin, naringin, p-coumaric acid, p-hydroxybenzoic acid, quercitrin, rutin, sinapic acid, and vanillic acid. Therefore, garlic leaves may serve as a valuable source of bioactive compounds, particularly during early spring. Studies have also shown that garlic leaves contain more vitamin C, a higher total polyphenol content, and exhibit stronger antioxidant activity than cloves [31]. Although garlic leaves are less commonly consumed in Europe and North America, they are widely used in Southeast Asia and China, especially in cuisines that emphasise fresh, seasonal ingredients [22,70].

According to published studies, substantial variability in polyphenol content has been reported among different garlic samples. For example, catechin ranged from not detected (N.D.) to 95.03 mg/kg, ellagic acid from N.D. to 103.50 mg/kg, hyperoside from 0.37 to 89.24 mg/kg, myricetin from 30.80 to 139.50 mg/kg, p-hydroxybenzoic acid from N.D. to 218.97 mg/kg, and rutin from 64.90 to 687.80 mg/kg. This variability likely reflects genetic differences among garlic cultivars. Additional contributing factors include soil composition, fertilisation practices, harvest maturity, postharvest storage, handling procedures, and climatic conditions [71,72].

**Table 2:** Summary of polyphenols detected in different garlic parts or processed forms.

Polyphenol Compounds	Range of Detection (mg/kg)	Type of Garlic	References
Acacetin	25.20	Raw garlic	[47]
Apigenin	3.24–23.20	Raw garlic	[51,73,74]
	N.D.	Black garlic	[22]
	17.90–38.80	Raw garlic leaves	[51]
β-Resorcylic acid	313.50–452.10	Raw garlic	[75]
Benzoic acid	N.D.–39.22	Raw garlic	[1,47]
Caffeic acid	0.06–81.97	Raw garlic	[1,22,47,51,73,74]
	12.21–25.58	Black garlic	[1,22]
	14.80–36.70	Raw garlic leaves	[51]
Catechin	N.D.–95.03	Raw garlic	[1,22,32,47,51]
	17.51	Black garlic	[22]
	5780.60	Raw garlic leaves	[51]
	1.12	Dried garlic	[76]
Catechol	9.53	Raw garlic	[47]
Chlorogenic acid	N.D.–93.94	Raw garlic	[1,22,47,51,73,74,77]
	13.40	Black garlic	[22]
	133.30–1068.70	Raw garlic leaves	[51]
Cinnamic acid	0.60	Raw garlic	[47]
Daidzein	N.D.–0.10	Raw garlic	[49,50]
Ellagic acid	14.29	Raw garlic	[47]
Epicatechin	N.D.–103.50	Raw garlic	[1,22,45,47,51,73,74,77]
	38.32	Black garlic	[22]
	42.60	Raw garlic leaves	[51]
Epigallocatechin	0.06	Raw garlic	[1]
Epigallocatechin gallate	0.55–1.03	Raw garlic	[1,22]
	19.52–23.12	Black garlic	[22]
Ferulic acid	0.06–39.66	Raw garlic	[22,47,51,73,74,77]
	31.00–138.50	Raw garlic leaves	[51]
Gallic acid	0.97–96.48	Raw garlic	[1,22,32,47,73,74,77]
	2.50–45.53	Black garlic	[1,22]
	7.74	Dried garlic	[76]
Genistein	0.10–0.20	Raw garlic	[49,50]
Hesperidin	N.D.–8.30	Raw garlic	[47,51]
	58.80–472.80	Raw garlic leaves	[51]
Hydroxytyrosol	0.16	Raw garlic	[47]
Hyperoside	0.37–89.24	Raw garlic	[73,74,77]
Isoferulic acid	2.78	Raw garlic	[47]
Isoorientin	0.91	Dried garlic	[76]
Isovanillic acid	3.02	Dried garlic	[76]
Kaempferol	0.07–23.90	Raw garlic	[22,47,51]
	N.D.	Black garlic	[22]
	7.90–26.60	Raw garlic leaves	[51]
	1.13	Dried garlic	[76]
Luteolin	0.15–22.92	Raw garlic	[51,73,74]
	9.30–46.70	Raw garlic leaves	[51]
m-Coumaric acid	4.84	Raw garlic	[22,74]
	13.99	Black garlic	[22]
Morin	1.06–1.33	Raw garlic	[22]
	6.19–7.74	Black garlic	[22]
Myricetin	30.80–139.50	Raw garlic	[51]
	81.40–105.30	Raw garlic leaves	[51]
Naringenin	N.D.–56.71	Raw garlic	[22,73,74]
	N.D.	Black garlic	[22]
Naringin	22.30–70.40	Raw garlic	[51]
	73.60–221.70	Raw garlic leaves	[51]
o-Coumaric acid	0.66	Raw garlic	[22,74]
	14.44	Black garlic	[22]
Orientin	0.95	Dried garlic	[32]
p-Coumaric acid	0.55–22.80	Raw garlic	[22,47,51,73,74]
	32.73	Black garlic	[22]
	29.50–106.0	Raw garlic leaves	[51]
p-Hydroxybenzoic acid	N.D.–218.97	Raw garlic	[51,73,74]
	125.50–914.80	Raw garlic leaves	[51]
Protocatechuic acid	4.22	Raw garlic	[47]
	2.23	Dried garlic	[32]
Pyrogallol	426.26	Raw garlic	[47]
Quercetin	N.D.–0.13	Raw garlic	[22,47]
	7.31	Black garlic	[22]
	0.56	Dried garlic	[76]
Quercitrin	0.14–14.52	Raw garlic	[22,47,51,73]
	9.89	Black garlic	[22]
	6.00–62.80	Raw garlic leaves	[51]
Resveratrol	0.58–0.61	Raw garlic	[22,47]
	6.42	Black garlic	[22]
Rosmarinic acid	0.31	Raw garlic	[47]
Rutin	N.D.–43.43	Raw garlic	[47,51,73,74]
	64.90–687.80	Raw garlic leaves	[51]
	0.10	Dried garlic	[76]
Salicylic acid	1.77	Raw garlic	[47]
Sinapic acid	1.00–5.40	Raw garlic	[51]
	139.60–233.90	Raw garlic leaves	[51]
Syringic acid	1.77–200.02	Raw garlic	[32,47]
Vanillic acid	N.D.–3.20	Raw garlic	[1,22,47,51,74]
	6.41	Black garlic	[22]
	22.60–64.80	Raw garlic leaves	[51]
Vitexin	1.98	Dried garlic	[76]

The table summarises the minimum and maximum amounts of polyphenolic compounds identified in various parts of garlic and its processed products. In cases where only a single reference reported the concentration of a specific compound, a single value is presented. All quantitative data originally expressed in parts per million (ppm), nmol/g, µg/g, or mg/100 g have been converted to mg/kg for consistency. In addition to bona fide phenolic compounds, the table also includes benzoic acid and cinnamic acid, which themselves are not phenolic acids but which serve as precursors of hydroxybenzoic and hydroxycinnamic acids, respectively—structural derivatives that represent major subclasses of natural phenolics. N.D., not detected.

Reported quercetin content in raw garlic rarely exceeds 0.13 mg/kg (Table 2). Considering typical garlic consumption (3–5 g/day), the estimated intake would provide <1 µg of quercetin daily, far below doses used in in vitro experiments (10–100 µM). This discrepancy highlights the gap between experimental exposure levels and realistic dietary intake.

## 3. Garlic Polyphenol Anticancer Properties

Polyphenols have potential both as chemopreventive and therapeutic agents, but there are significant challenges in definitively establishing their benefits in humans, among other things, due to low bioavailability, short half-life, and in vivo metabolism, which makes it difficult to distinguish their effects in prevention from their effects in cancer therapy [78]. The majority of the evidence discussed in this section is derived from in vitro studies using established cancer cell lines. In vivo data are available for selected compounds, whereas clinical evidence in humans remains limited and inconclusive. Therefore, the proposed mechanisms should be interpreted primarily as preclinical observations rather than clinically validated pathways. Cancer remains one of the most pressing health challenges in modern society. Consequently, many dietary polyphenols have been investigated for anticancer activity. When focusing on phenolic constituents reported in garlic, the body of evidence is predominantly in vitro (Table 3). Some compounds included in Table 3—notably resveratrol, and luteolin—have, however, been investigated extensively in tumour-bearing animal models. For most of the remaining compounds, in vivo studies are still limited and human data remain scarce. According to the 2022 World Health Organization (WHO) report, the three most prevalent cancer types worldwide are lung, breast, and colorectal cancers [25]. It is therefore not surprising that most in vitro studies have focused on cell lines derived from these cancer types. Current in vitro evidence indicates that many polyphenols identified in garlic exhibit antiproliferative or pro-apoptotic activity in cancer cell models (Table 3). The table summarises representative publications describing the in vitro anticancer effects of garlic-derived polyphenols.

Most in vitro studies investigating garlic-derived polyphenols employ concentrations ranging from 10 to 200 µM, frequently exceeding plasma levels achievable through dietary intake. For example, peak plasma concentrations of quercetin after oral administration typically remain below 5 µM, often in the low micromolar or submicromolar range (0.3–2 µM) depending on dose and formulation [152,153]. Similarly, resveratrol plasma levels rarely exceed 2 µM following oral intake due to rapid metabolism [154]. Moreover, most mechanistic studies are conducted in 2D monoculture systems, which do not replicate tumour microenvironment complexity, stromal interactions, or pharmacokinetic constraints. In many cases, exposure times (24–72 h) and single-dose designs may overestimate cytotoxic effects compared with dynamic in vivo conditions. Therefore, while in vitro findings provide valuable mechanistic insights, the concentrations required for robust cytotoxicity are often supraphysiological, limiting direct translational interpretation.

The literature indicates that polyphenols identified in garlic have been primarily investigated in cell lines derived from the digestive system (e.g., CACO-2, HT-29, HCT-15, HCT-116, SW480), breast cancers (e.g., MCF-7, MDA-MB-231, 4T1, BT474, MCF10A, SK-BR-3), and lung cancers (e.g., A549, H1299) [80,81,82,85,87,101]. These cancer types correspond to the most prevalent cancers identified by the WHO (summarised in Table 3). However, other cancer types have also been studied in the context of polyphenols derived from plants, including garlic. The most commonly investigated cancers beyond these include those of the nervous system (e.g., U118, U87MG, SH-SY5Y, U251, U343), liver (e.g., HepG2, Hep3B), and prostate (e.g., DU145, PC3, LNCaP) (summarised in Table 3) [82,83,85,86,97,98]. In the cell lines mentioned, similar mechanisms of action for the studied polyphenols have been observed. These include inhibition of cell proliferation, a decrease in cell viability, and, in most cases, initiation of the apoptosis process [82,83,91,92,122,123] (summarised in Table 3). In the vast majority of studied cell lines, the described polyphenols selectively induce cell death in cancerous cells without causing significant damage to normal cells. Moreover, research consistently shows that apoptosis is mediated by an increased level of reactive oxygen species (ROS) [82,83,89,90,106,107].

The anticancer effect of individual polyphenols has also been studied in animal models. Researchers demonstrated that, in a rat oesophageal carcinogenesis model induced by N-nitrosobenzylmethylamine (NBMA), dietary administration of ellagic acid at concentrations of 0.4 and 4 g/kg diet significantly decreased the average number of tumours by 21–55% after 20 and 27 weeks of exposure [155]. This study was conducted using rats receiving ellagic acid incorporated into a semi-purified diet, representing a long-term chemoprevention model rather than acute pharmacological treatment. Additionally, ellagic acid inhibited both preneoplastic and neoplastic lesions in this bioassay [155]. It was shown that ellagic acid reduces the formation of NBMA metabolites as well as their binding to DNA in cultured rat oesophageal explants [156]. However, the potential use of ellagic acid as a cancer chemopreventive agent is limited by its poor bioavailability when administered orally and its low solubility in water [157,158]. This limitation was overcome by administering ellagic acid as a subdermal implant, which was evaluated in oestrogen-induced rat mammary tumours. Notably, a much smaller dose of ellagic acid was required via subdermal administration to achieve a similar reduction in tumour burden, compared with dietary intake [159]. Mechanistically, in terms of its cancer chemopreventive potential, ellagic acid modulates various cell regulatory proteins. This includes the downregulation of p-STAT3, p-Akt, and p-ERK1/2, which leads to cell apoptosis or inhibition of proliferation [157,160].

In another study, resveratrol inhibited the development of preneoplastic lesions in carcinogen-treated mouse mammary glands in culture. In mouse skin carcinogenesis models induced by DMBA/TPA, resveratrol treatment significantly reduced tumour incidence, multiplicity, and tumour volume by promoting apoptosis and suppressing tumour growth, demonstrating chemopreventive efficacy in vivo [161]. Additionally, a separate study demonstrated the antioxidant activity of resveratrol in preventing tumour initiation. This was achieved by inhibiting angiogenesis through interactions with vascular endothelial growth factor (VEGF) and metalloproteases [46]. In vivo experiments demonstrated that resveratrol inhibited tumour growth in prostate cancer xenograft models. For example, oral administration of resveratrol at 30 mg/kg/day significantly reduced tumour volume, angiogenesis, and proliferation in prostate cancer xenograft mice, accompanied by increased apoptosis and decreased neovascularization [162]. Resveratrol also inhibited tumour progression in transgenic adenocarcinoma of the mouse prostate (TRAMP) models, where dietary administration reduced tumour incidence, delayed tumour onset, and decreased tumour cell proliferation [163].

To date, animal and human studies on the anticancer effects of garlic have primarily focused on sulphur compounds. However, the relationship between organic sulphur compounds and phenolic compounds, as well as their combined biological activity, remains poorly understood. Researchers emphasise the synergistic therapeutic effects of these compound classes [164]. Li et al. demonstrated that daily intraperitoneal administration of raw garlic extract in C57BL/6 mice inoculated with 2 × 10^6^ EL4 lymphoma cells resulted in complete tumour regression [165]. Each mouse received 1 mL RGE, equivalent to approximately 100 mg wet weight of raw garlic (≈6.7 g/kg body weight), administered once daily for 21 consecutive days. This treatment completely prevented ascites formation and resulted in long-term survival without detectable adverse effects, highlighting the potent anticancer efficacy of directly administered garlic extract in vivo [165]. Other studies have shown that supplementation with raw garlic powder reduced the incidence of mammary cancer in rats and prevented mammary carcinogenesis induced by N-methyl-N-nitrosourea [166,167]. Unfortunately, most available studies focus on sulphur compounds. These have been shown to mildly reduce tumour incidence and severity in models of N-nitroso compound-induced carcinogenesis [168]. This reductionist approach has led to numerous studies evaluating individual garlic constituents for their anticancer properties. In most animal studies, oral administration of garlic or its isolated compounds resulted in weak direct anticancer effects at best [169]. These and other findings suggest that garlic’s anticancer properties may be more effective upon direct exposure to cancer cells, rather than after absorption through the gastrointestinal epithelium. Moreover, it cannot be excluded that garlic polyphenols exert their strongest effects as a mixture, where compound interactions enhance bioactivity.

Most human studies on garlic’s anticancer effects have been retrospective surveys, aimed at identifying potential associations between cooked garlic consumption and cancer incidence or progression [170,171,172]. Some studies have involved dietary interventions in which participants consumed garlic. However, direct evidence supporting garlic’s anticancer potential in humans remains weak [173,174]. Studies specifically addressing polyphenol effects in humans are particularly rare. In clinical trials on healthy volunteers, Goldberg et al. investigated the absorption kinetics of resveratrol, quercetin, and catechin after ingestion [175]. Their results showed that peak plasma concentrations occurred at 30 min post-consumption. However, levels returned to baseline within 4 h [175]. Further research by Walle et al. and Goldberg et al. confirmed the low bioavailability of these polyphenols in their active forms [175,176]. Therefore, to exert sustained biological effects, polyphenols would require continuous dietary intake. Currently, it is believed that regular garlic consumption, as part of a balanced diet, may support cancer prevention and therapy [16]. However, it is important to emphasise that garlic provides supportive effects only and does not replace conventional cancer treatments. The relationship between polyphenol intake and cancer risk has been regularly assessed and meta-analysed over the past 10–15 years. For example, a meta-analysis of prospective studies found that isoflavone consumption was associated with a 19% reduction in gastric cancer risk [177]. Despite decades of research, the anticancer properties of garlic still lack conclusive evidence, particularly regarding curative effects against aggressive cancers in animal models or humans.

Most garlic-derived polyphenols undergo extensive phase II metabolism, including glucuronidation, sulfation, and methylation in the intestinal epithelium and liver [178]. These conjugated metabolites often exhibit altered biological activity compared with parent compounds. Importantly, the gut microbiota play a central role in determining the metabolic fate and biological activity of polyphenols. A substantial proportion of ingested polyphenols is poorly absorbed in the small intestine and therefore reaches the colon, where polyphenols undergo extensive microbial biotransformation [179]. Through enzymatic processes such as dehydroxylation, decarboxylation, and ring cleavage, gut microorganisms generate smaller phenolic metabolites that often differ in bioavailability, stability, and biological activity compared with the original compounds [180]. In many cases, these microbial metabolites exhibit increased systemic availability and may contribute significantly to the biological effects attributed to dietary polyphenols. The composition, metabolic capacity, and functional activity of the gut microbiota are therefore critical determinants of polyphenol metabolism and systemic exposure, which may partly explain the substantial inter-individual variability observed in experimental and clinical outcomes [181]. Moreover, the interaction between polyphenols and the microbiota is bidirectional. Polyphenols can modulate the composition and metabolic activity of the microbiota, whereas the microbiota determine the profile and abundance of bioactive polyphenol metabolites, thereby influencing their physiological effects in the host [182]. In addition, factors such as the food matrix, co-consumed nutrients, inter-individual differences in microbiota composition, and genetic polymorphisms affecting metabolic enzymes further modulate polyphenol bioavailability and biological activity.

It should also be acknowledged that positive findings may be overrepresented in the literature. Null or weak effects of polyphenols are less frequently published, potentially contributing to publication bias. Furthermore, some studies report only modest reductions in cell viability or inconsistent pro-apoptotic responses depending on cell type and genetic background. This variability suggests that the anticancer effects of garlic-derived polyphenols are context-dependent and strongly influenced by metabolic and microbiome-related factors rather than being universal.

## 4. The Common Mechanism of Action of Polyphenols Found in Garlic

**Figure 1:**
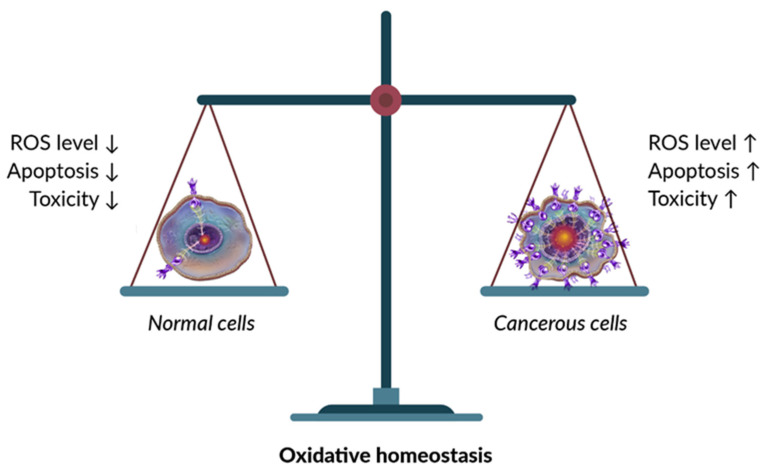
Impact of garlic polyphenols on oxidative homeostasis. In normal cells, mild oxidative stress triggered by garlic polyphenols reduces intracellular ROS levels, decreases apoptosis, and protects against toxicity induced by damaging agents. Conversely, in cancer cells, the same polyphenols enhance ROS generation, promote apoptosis, and induce cytotoxic effects. These differential effects on redox homeostasis and cell survival are well established and widely supported by experimental evidence reported in the scientific literature.

The high polyphenol content in garlic is commonly associated with strong antioxidant and health-promoting properties. This is largely attributed to their ability to scavenge endogenous ROS. Numerous studies on garlic extracts have shown that high polyphenol levels protect normal cells from ROS-dependent cell death [183,184]. For instance, aged garlic extract was reported to reduce ROS-dependent cell death in SH-SY5Y cells—a model of human neuroblasts—by activating Nrf2 [183]. Similarly, reduced oxidative stress via Nrf2 activation has been observed in male Sprague–Dawley rats fed a 65% fructose diet and receiving raw garlic homogenate (250 mg/kg/day) for 8 weeks [185]. It is well-documented that polyphenols such as chlorogenic acid, resveratrol, quercetin, ferulic acid, and rosmarinic acid activate Nrf2 [186]. However, depending on their type and concentration, some polyphenols may also inhibit Nrf2 activity [187]. Nrf2 binds to the antioxidant response element (ARE) and plays a key role in regulating the expression of various detoxifying and antioxidant genes, including heme oxygenase-1 (HO-1). It also controls enzymes such as superoxide dismutase (SOD), catalase (CAT), and glutathione peroxidase (GPx) [188]. Increased expression of these enzymes protects normal cells from oxidative stress and ROS-induced apoptosis. In contrast, in cancer cells, high levels of polyphenols often increase toxicity [189]. Plant-derived polyphenols have been reported to exert pro-oxidant effects through interactions with Nrf2, which may contribute to their anticancer and apoptosis-inducing properties [190]. Many of the studies summarized in Table 3 describe the anticancer activity of garlic-derived polyphenols by comparing their effects on cancerous and normal cells. These studies generally show that cancer cells exhibit higher basal ROS levels. An additional increase in ROS due to the oxidative action of polyphenols or their interaction with Nrf2 may drive ROS-dependent cell death [191]. This elevated ROS level in cancer cells reflects an imbalance between oxidants and antioxidants. Treatment with polyphenols can further modulate molecular pathways, amplifying their anticancer effects. In normal cells, polyphenols usually cause only a modest increase in ROS production. However, this is accompanied by upregulation or activation of antioxidant enzymes, ultimately providing long-term protection (Figure 1).

Peroxisome proliferator-activated receptors (PPARs) are ligand-activated transcription factors belonging to the nuclear hormone receptor superfamily. They comprise three subtypes: PPARα, PPARβ/δ, and PPARγ [192]. PPARs regulate energy metabolism in various tissues, including the liver, adipose tissue, and muscle [193]. All PPAR isoforms modulate lipid accumulation, cell differentiation, and apoptosis in cancer cells [194,195]. Additionally, PPARs can act as receptors for environmental factors, including dietary polyphenols [73]. Several phenolic acids, such as gentisic, p-hydroxybenzoic, chlorogenic, caffeic, p-anisic, ferulic, and gallic acid, as well as flavonoids like hesperidin, naringin, and epicatechin, have been shown to increase PPARα mRNA expression [196]. In contrast, compounds such as epicatechin, epicatechin-3-gallate, epigallocatechin, epigallocatechin-3-gallate, catechin, and gallocatechin increase PPARγ mRNA expression, while exhibiting little or no effect on PPARα [197]. Moreover, these flavonoids also function as partial PPARγ agonists [198]. To date, several polyphenols listed in Table 2—including luteolin, quercetin, p-coumaric acid, kaempferol, apigenin, ferulic acid, and ellagic acid—have been described as directly interacting with PPARγ and influencing the differentiation of mouse preadipocytes in the 3T3-L1 cell line [73]. Interestingly, some flavonoids such as quercetin, kaempferol, and resveratrol act as agonists of all PPAR subtypes and affect both their expression and activity [199,200,201,202]. All PPARs influence ROS homeostasis and exert antioxidant effects by regulating the transcription of endogenous antioxidant genes. Additionally, they coordinate intracellular signalling pathways that reduce ROS production, either directly or indirectly [203]. Numerous studies have shown that ligands of PPARα, PPARβ/δ, and PPARγ enhance the expression and activity of catalase (CAT) [203]. Furthermore, other enzymes involved in ROS homeostasis—including SOD1, SOD2, GPx, and HO-1—are also regulated by PPAR ligands [203,204]. Garlic-derived polyphenols have been shown to increase the expression and/or activity of antioxidant enzymes. In a previous study, our team demonstrated that the Spanish garlic cultivars Morado and Castano exhibited the highest total polyphenol content among the analyzed garlic varieties. This high polyphenol content was correlated with increased toxicity in human skin fibroblast (BJ) cells [32]. Further research revealed that the Morado cultivar induced ROS-dependent cell death in the human squamous carcinoma (SCC-15) cell line [184]. In our most recent study, we showed that Morado also activated both mRNA and protein expression of PPARγ, along with upregulation of SOD1 and CAT [205].

**Figure 2:**
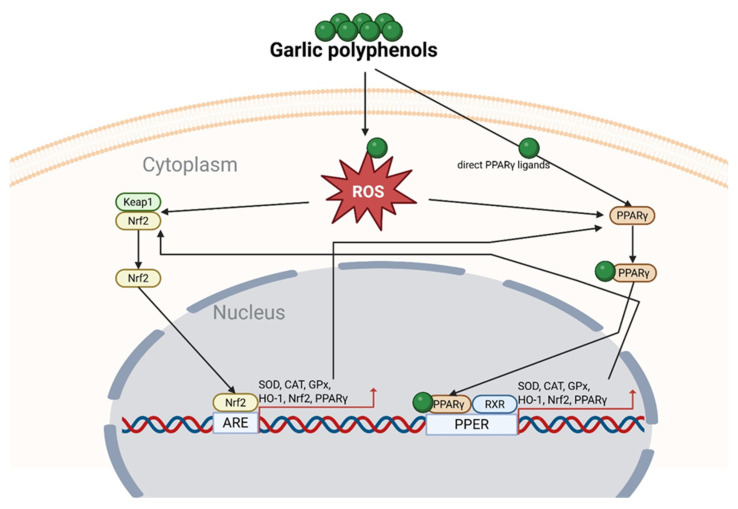
Hypothetical integrative model of Nrf2–PPARγ crosstalk in the anticancer activity of garlic-derived polyphenols (based primarily on in vitro evidence). Garlic polyphenols affect both Nrf2 and PPARγ pathways, regulating detoxification, cell apoptosis, differentiation, and oxidative stress homeostasis. Both Nrf2 and PPARγ influence antioxidant enzymes such as SOD, CAT, GPX, and HO-1. Furthermore, an ARE has been identified in the PPARγ promoter, and PPREs have been found in the Nrf2 promoter region, resulting in cross-talk between these receptors. Abbreviations: CAT—catalase; GPx—glutathione peroxidase; HO-1—heme oxygenase-1; Nrf2—nuclear factor erythroid 2-related factor 2; PPARγ—peroxisome proliferator-activated receptor gamma; SOD—superoxide dismutase.

A functional interaction between the Nrf2 and PPARγ signalling pathways has also been reported [206]. An antioxidant response element (ARE) has been identified within the PPARγ promoter, while peroxisome proliferator response elements (PPREs) have been detected in the Nrf2 promoter region [207]. The presence of these response elements suggests a positive feedback loop between the Nrf2 and PPARγ pathways. This crosstalk may facilitate mutual upregulation of both transcription factors and their downstream antioxidant targets [206,208]. Based on currently available preclinical evidence, we propose a hypothetical model in which crosstalk between Nrf2 and PPARγ may contribute to the anticancer activity of garlic-derived polyphenols (Figure 2). However, direct experimental confirmation of this interaction in cancer models remains limited. While many of these compounds share common mechanisms of action, individual polyphenols may also exert distinct, compound-specific effects, as described in the following chapter.

Garlic-derived polyphenols exhibit a dual influence on the Nrf2/ARE oxidative stress pathway. Many garlic polyphenols are Nrf2 activators, inducing antioxidant defences—for example, chlorogenic acid, resveratrol, quercetin, ferulic acid, and rosmarinic acid are well-documented as activating Nrf2 [186]. This leads to upregulation of detoxifying enzymes such as e.g., HO-1, SOD, CAT, GPx, which helps protect normal cells from oxidative damage [185]. Conversely, some polyphenols can inhibit Nrf2 activity depending on compound and dose [187]. In cancer cells—which often have elevated basal ROS, high levels of polyphenols frequently act as pro-oxidants, overwhelming the cells’ antioxidant defences [190]. Such polyphenols may suppress Nrf2 or otherwise interfere with its signalling, resulting in excessive ROS accumulation and oxidative stress-induced apoptosis in tumour cells [191]. This context-dependent modulation of Nrf2 helps explain how garlic polyphenols can safeguard healthy cells via antioxidant pathways yet promote cytotoxic oxidative stress in cancer cells, contributing to their anticancer efficacy.

A major anticancer mechanism of garlic polyphenols is the induction of oxidative stress in cancer cells leading to apoptosis. Numerous studies show that these compounds selectively elevate ROS levels in tumour cells, driving ROS-mediated cytotoxicity [191]. For instance, in vitro experiments with garlic’s phenolics such as e.g., caffeic, chlorogenic, p-coumaric, gallic acids, consistently report a significant increase in intracellular ROS in cancer cell lines [89,93]. This usually leads to apoptosis often accompanied by activation of caspases and DNA damage [84]. The excess ROS generated by polyphenols overwhelms the antioxidant defences of cancer cells, resulting in oxidative injury to critical biomolecules. In the case of gallic acid exposure of cancer cells leads to the generation of semiquinone radicals and H_2_O_2_ that raise ROS to cytotoxic levels, causing oxidative DNA damage and mitochondrial dysfunction [209]. This oxidative surge triggers the mitochondrial (intrinsic) apoptosis pathway: cancer cells treated with gallic acid (and other similar polyphenols) show collapse of mitochondrial membrane potential, lipid peroxidation, Bax upregulation/Bcl-2 downregulation, cytochrome c release, and activation of caspases-9 and -3 [107]. Collectively, such findings indicate that garlic polyphenols push cancer cells past their redox threshold, inducing apoptosis via ROS-dependent mechanisms. Importantly, this pro-oxidant effect tends to be selective for malignant cells. Normal cells experience only a mild ROS increase alongside enhanced antioxidant enzyme expression, which ultimately protects them from damage [210]. This differential outcome underlies the therapeutic window of garlic polyphenols, allowing them to kill cancer cells through oxidative stress while sparing healthy tissue.

## 5. Detailed Mechanisms of Action of Selected Garlic-Derived Polyphenols

Garlic-derived polyphenols exert common anticancer effects by engaging a network of cellular pathways that regulate oxidative stress, inflammation, proliferation, and cell death. However, each polyphenol also possesses distinct molecular targets and potencies. Below, we discuss the key mechanisms and cancer-related pathways modulated by six prominent garlic polyphenols—quercetin, kaempferol, caffeic acid, ferulic acid, p-coumaric acid, and gallic acid—highlighting both their shared pathways and unique features.

**Figure 3:**
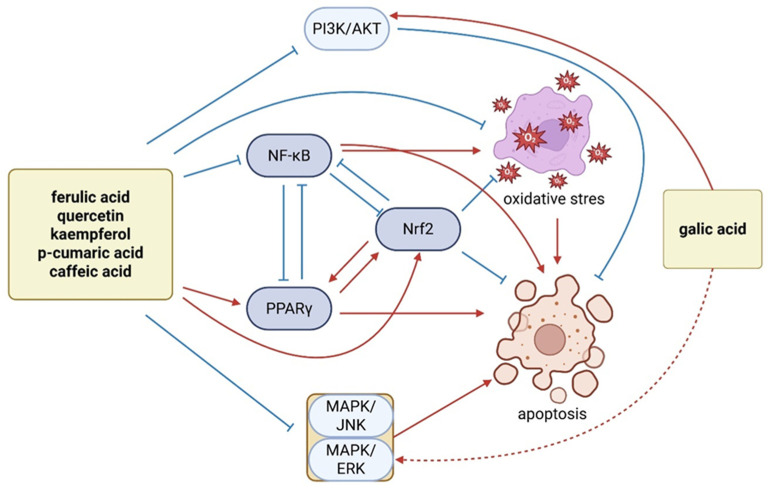
Mechanisms of action of the crucial polyphenols. The diagram integrates mechanisms demonstrated in vitro and in selected in vivo models. Dashed lines indicate interactions that remain hypothetical or inferred from related polyphenols rather than directly demonstrated for garlic-derived compounds. The dashed line indicates a potential effect, red lines indicate stimulation, and blue lines indicate inhibition. Abbreviations: ARE—Antioxidant Response Element; Bax/Bcl-2—Bcl-2-associated X protein/B-cell lymphoma 2; ERK—extracellular signal-regulated kinase; JNK—c-Jun N-terminal kinase; MAPK—Mitogen-Activated Protein Kinase; NF-κB—Nuclear Factor kappa-light-chain-enhancer of activated B cells; Nrf2—Nuclear factor erythroid 2–related factor 2; PI3K/Akt—Phosphoinositide 3-kinase/Protein kinase B; PPARγ—peroxisome proliferator-activated receptor gamma.

To highlight these differences and similarities, Table 4 summarises the major garlic-derived polyphenols along with their proposed anticancer mechanisms. Importantly, the overall anticancer activity of garlic is likely mediated by the synergistic interaction of its multiple polyphenolic constituents. Therefore, the extent and nature of its biological effects may vary depending on factors such as garlic cultivar, cultivation conditions, and post-harvest processing methods. The main mechanisms of action of the following polyphenols have been compiled and presented in Figure 3.

An important caveat is the poor bioavailability of many garlic-derived polyphenols [220]. For instance, quercetin’s peak plasma concentration is reached only transiently after ingestion, and levels return to baseline within 4 h [221]. Ferulic acid and other phenolics are also rapidly metabolized and excreted. Consequently, the effective doses observed in vitro or in animal models would be challenging to achieve through diet alone. Such pharmacokinetic limitations must be considered when translating these findings to humans.

### 5.1. Quercetin

Quercetin is a flavonol that robustly modulates multiple signalling pathways to inhibit tumour growth. A wealth of studies indicates quercetin can suppress cancer cell proliferation and induce apoptosis by interfering with the PI3K/Akt pathway and downstream transcription factors like NF-κB and STAT3 [222,223]. Through inhibition of NF-κB, quercetin downregulates the expression of anti-apoptotic and pro-inflammatory genes, thereby sensitizing cells to cell death signals [224]. Concurrently, quercetin activates the Nrf2/ARE pathway, leading to increased expression of cytoprotective antioxidant enzymes such as HO-1 and SOD [225]. This dual action—dampening NF-κB while boosting Nrf2—skews cells away from a pro-survival, pro-oxidant state and towards an environment hostile to cancer growth [210]. Mechanistic studies in lung, colon, prostate, and other tumour models have confirmed quercetin’s capacity to induce cell cycle arrest (often at G0/G1 or G2/M) and trigger intrinsic apoptosis via modulation of Bcl-2 family proteins [226]. For instance, treatment of colon carcinoma cells with quercetin elevates the Bax/Bcl-2 ratio, promoting mitochondrial cytochrome c release and caspase-3 activation [227]. In prostate cancer and leukemia cells, quercetin was shown to reduce cell viability and NF-κB activity, leading to increased apoptotic markers [60]. In vivo studies further underscore quercetin’s anticancer efficacy. In vivo, quercetin administered at 200 mg/kg significantly induced tumour cell apoptosis via caspase-3 activation in mice bearing Ehrlich ascites carcinomas, demonstrating selective anticancer activity without significant toxicity to normal tissues [228]. Taken together, quercetin’s anticancer activity stems from its multi-targeted modulation of survival pathways (PI3K/Akt, NF-κB), stress responses (Nrf2/ARE), and apoptotic regulators (Bax/Bcl-2, caspases), yielding a potent anti-proliferative and pro-apoptotic effect across diverse cancer types. Most of these mechanisms have been demonstrated in vitro, with selected confirmation in animal models; however, clinical validation is currently lacking.

### 5.2. Kaempferol

Kaempferol, another major flavonol in garlic, shares several anticancer mechanisms with quercetin but also exhibits distinct effects for specific pathways. Like quercetin, kaempferol inhibits the PI3K/Akt kinase cascade, leading to downstream suppression of NF-κB and other pro-survival signals [229]. Kaempferol treatment consistently increases expression of the tumour suppressor tensin homolog deleted on chromosome 10 (PTEN) in various models, which further antagonizes PI3K/Akt signalling and facilitates apoptotic onset [230]. Through NF-κB inhibition, kaempferol attenuates the production of inflammatory mediators (tumour necrosis factor alpha (TNF-α), interleukin-1 beta (IL-1β), cyclooxygenase-2 (COX-2)) that can fuel tumour progression [231]. In parallel, kaempferol can activate Nrf2 and upregulate ARE-driven genes, contributing to its antioxidant and cytoprotective profile [232]. A notable feature of kaempferol is its impact on mitogen-activated protein kinase (MAPK) pathways: it tends to inhibit mitogenic ERK signalling while it may activate stress-related MAPKs (JNK/p38) in a context-dependent manner [233]. In lung cancer models (A549 cells), kaempferol was found to inhibit Akt phosphorylation and enhance MEK/ERK activity as a necessary step for full caspase-7 activation—an intriguing crosstalk wherein a transient MAPK activation contributed to apoptosis induction [234]. Kaempferol also exerts antimetastatic effects by targeting extracellular proteases and motility pathways. It downregulates matrix metalloproteinases 2 and 9 (MMP-2 and MMP-9) expression, enzymes critical for tumour invasion, and can interfere with transforming growth factor beta (TGF-β)/Smad signalling to prevent epithelial–mesenchymal transition (EMT) [235]. In vivo, kaempferol has demonstrated the ability to reduce metastasis and tumour growth. In a B16 melanoma mouse model, kaempferol significantly decreased pulmonary metastatic nodules, correlating with reduced MAPK/PKC signalling and MMP-9 activity in the tumours [236]. Moreover, kaempferol can synergize with chemotherapeutics; co-treatment of colon cancer xenografts with kaempferol and fluorouracil (5-FU) enhanced tumour regression via augmented Akt inhibition and apoptosis [237]. In summary, kaempferol broadly converges on the same anti-inflammatory and pro-apoptotic pathways as quercetin (notably NF-κB and Akt suppression), but also distinctly impacts cell migration and cell cycle regulators (e.g., CDKs and p53-dependent pathways), underscoring its multi-faceted anticancer profile.

### 5.3. Caffeic Acid

Caffeic acid is a hydroxycinnamic acid that contributes to garlic’s antioxidant and anticancer properties. A primary mode of action of caffeic acid is the mitigation of oxidative stress: it is a well-known ROS scavenger and also an inducer of the Nrf2/ARE pathway [238]. By stabilizing Nrf2, caffeic acid increases cellular glutathione and detoxifying enzymes, which protect normal cells from carcinogenic insults and oxidative DNA damage. In parallel, caffeic acid inhibits major pro-tumourigenic signalling pathways. It has been shown to block the activation of NF-κB, resulting in lowered expression of NF-κB target genes such as vascular endothelial growth factor (VEGF) (a key angiogenic factor) and MMP-9 (a metastasis-promoting protease) in hepatocellular carcinoma models [239]. This corresponds with a reduction in tumour angiogenesis and invasiveness. Additionally, caffeic acid can interfere with the MAPK cascade; for example, in skin cancer cells, it significantly reduced phosphorylation of ERK1/2, JNK, and p38 MAPK, thereby disrupting mitogenic and stress signalling needed for tumour growth [240]. Through inhibition of both NF-κB and MAPKs, caffeic acid exerts an anti-inflammatory effect that is linked to decreased production of IL-6, IL-1β, and COX-2 in the tumour microenvironment [241]. On the PI3K/Akt axis, caffeic acid was found to decrease phosphorylation of Akt and its downstream effector mTOR, contributing to growth arrest and autophagic cell death in certain cancer cell lines [242]. At higher concentrations, caffeic acid directly induces mitochondrial dysfunction—it can depolarize the mitochondrial membrane (loss of ΔΨm) and elevate intracellular ROS within cancer cells, leading to apoptosis via the intrinsic pathway [243]. Indeed, treatment of hepatoma cells with caffeic acid triggered cytochrome c release and caspase-9/-3 activation, consistent with mitochondrial apoptotic induction [244]. Caffeic acid has shown chemopreventive efficacy in vivo as well. Low-dose caffeic acid (and its derivative caffeic acid phenethyl ester) caused complete regression of implanted hepatomas in mice, an effect attributed to a “dual mechanism” of NF-κB inhibition and pro-oxidant DNA damage in tumour cells [245]. Animal models of skin cancer and colon cancer have similarly noted that dietary caffeic acid can reduce tumour incidence and multiplicity, accompanied by lower inflammatory markers and increased antioxidant enzyme levels in tissues [246]. Collectively, caffeic acid’s anticancer mechanisms center on reducing oxidative and inflammatory stimuli (via Nrf2 activation and NF-κB inhibition) and directly promoting apoptotic pathways, making it an effective agent in both cancer prevention and therapy.

### 5.4. Ferulic Acid

Ferulic acid, another abundant hydroxycinnamic acid in garlic, is distinguished by its potent antioxidant action and ability to modulate cell cycle regulators. Ferulic acid strongly activates the Nrf2/ARE pathway; in fact, it can upregulate Nrf2 and its target genes to a degree comparable to classic chemopreventive agents, thereby fortifying cells’ antioxidant defences [247]. It also engages PPARγ, a nuclear receptor involved in cellular metabolism and differentiation: ferulic acid treatment has been shown to increase PPARγ expression and activity in certain models, an effect linked to anti-inflammatory outcomes via repression of NF-κB [248]. Through PPARγ activation, ferulic acid may promote a more differentiated, less proliferative state in tumour cells and enhance lipid metabolic reprogramming unfavorable to rapid growth [249]. Like other polyphenols, ferulic acid inhibits the NF-κB pathway; for instance, in LPS-stimulated macrophages, ferulic acid prevented IκBα degradation and nuclear p65 translocation, thereby blocking NF-κB–mediated transcription of IL-6 and TNF-α [250]. In cancer cells, this NF-κB inhibition translates to lower expression of survival proteins (e.g., Bcl-xL, survivin) and reduced chronic inflammation that can drive tumour progression. Ferulic acid also targets the PI3K/Akt pathway. In cervical carcinoma cells, ferulic acid was observed to markedly reduce phosphorylated Akt levels and downstream kinase signals, coinciding with G0/G1 cell cycle arrest [214]. The cell cycle blockade by ferulic acid is attributed to downregulation of cyclins (Cyclin D1, E) and CDKs alongside upregulation of CDK inhibitors like p21Cip1 [214,251]. Such changes halt the transition from G1 to S phase, giving ferulic acid a strong antiproliferative effect. On triggering apoptosis, ferulic acid utilizes both intrinsic and extrinsic pathways. It can increase pro-apoptotic Bax and decrease anti-apoptotic Bcl-2, tilting the balance toward mitochondrial apoptosis [214]. In HeLa and CaSki cervical cancer cells, ferulic acid induced DNA fragmentation and PARP-1 cleavage, hallmarks of apoptosis, and these effects were accompanied by activation of caspase-3 [252,253]. Interestingly, ferulic acid may also interfere with growth factor signalling; a study by Yang et al. (2015) reported that ferulic acid suppressed fibroblast growth factor-1 (FGF1) and its receptor FGFR1 in melanoma cells, leading to downstream inhibition of the PI3K/Akt pathway and inhibited tumour cell migration [254]. In vivo, ferulic acid displays chemopreventive and therapeutic properties. In male Wistar rats with hepatocellular carcinoma induced using N-nitrosodiethylamine (NDEA; 200 mg/kg, i.p.) and carbon tetrachloride (CCl_4_; 0.5 mL/kg), ferulic acid treatment at 25 or 50 mg/kg was associated with upregulation of hepatic Nrf2 and p53 and downregulation of Akt/PKB–NF-κB–TNF-α signalling, correlating with reduced disease burden. [255] Ferulic acid has also been shown to protect normal tissues from chemotherapy-induced damage (e.g., nephrotoxicity) by activating Nrf2/HO-1 and PPARγ in vivo, all the while sensitizing tumour cells to the chemotherapy via NF-κB suppression [256]. In summary, ferulic acid’s anticancer mechanism is multi-pronged: it restrains cell proliferation through cell cycle arrest, promotes apoptosis by modulating Bcl-2 family proteins and caspases, and attenuates inflammation and oxidative stress via NF-κB inhibition and Nrf2/PPARγ activation. These properties make it a compelling adjunct in cancer prevention and in enhancing the efficacy of conventional therapies.

### 5.5. p-Coumaric Acid

p-Coumaric acid is a phenolic acid that, although less studied than ferulic or caffeic acid, has shown notable anti-cancer effects, particularly in colorectal cancer models. Mechanistically, p-coumaric acid powerfully activates the Nrf2 pathway, which is thought to underlie its chemopreventive activity in the colon [257]. In a short-term carcinogen model, Sharma et al. (2019) found that p-coumaric acid supplementation induced nuclear translocation of Nrf2 in colon mucosal cells, elevating phase II enzyme expression and significantly reducing early precancerous lesions [257]. This Nrf2-driven antioxidant response protects cells from DNA damage and also indirectly reduces NF-κB activity (as Nrf2 and NF-κB can negatively regulate each other). Indeed, an anti-inflammatory role of p-coumaric acid is well documented: it inhibits NF-κB activation, resulting in decreased levels of pro-inflammatory cytokines and mediators (IL-6, IL-1β, COX-2) in vitro [258]. By impairing NF-κB, p-coumaric acid also diminishes expression of cyclin D1 and other proliferation drivers, contributing to cell cycle arrest in cancer cells [259]. A distinct feature of p-coumaric acid is its ability to induce endoplasmic reticulum (ER) stress and the unfolded protein response (UPR) in tumour cells. Treatment of colon cancer cells with p-coumaric acid was shown to downregulate the chaperone GRP78 and activate PERK-eIF2α signalling, leading to C/EBP homologous protein (CHOP)-mediated apoptosis [215]. This links p-coumaric acid to the disruption of proteostasis in cancer cells, a stress that can selectively kill malignant cells laden with misfolded proteins. On the mitochondrial apoptosis front, p-coumaric acid increases the Bax/Bcl-2 ratio and elevates Cyt-c release, effectively engaging the intrinsic apoptotic pathway [216]. Caspase-9 and -3 activation has been observed following p-coumaric acid treatment in breast and colon cancer cell lines, confirming execution of apoptosis [216]. Additionally, p-coumaric acid can inhibit growth factor signalling such as IGF-1R/Akt, and it modestly suppresses MAPK/ERK activity, thereby blocking proliferative and survival cues [260,261]. In vivo studies reinforce these findings: p-coumaric acid administration in rats attenuated dimethylhydrazine-induced colon carcinogenesis, with treated animals showing lower NF-κB and higher Nrf2 activity in colonic tissues compared to controls [257]. The treated rats also exhibited reduced inflammatory cell infiltration and a decline in dysplastic aberrant crypt foci, indicating a chemo-preventive effect via modulation of the Nrf2–NF-κB axis. Beyond the colon, p-coumaric acid has demonstrated anti-neoplastic effects in models of skin and liver cancer, again correlating with enhanced antioxidant enzyme levels and reduced oxidative damage [262]. In summary, p-coumaric acid combats cancer through antioxidant activation (Nrf2), inflammation inhibition (NF-κB), and pro-apoptotic stress induction (ER stress and mitochondrial pathways). Its capacity to simultaneously trigger protective responses in normal cells and lethal stress in cancer cells exemplifies the nuanced chemopreventive potential of dietary phenolics. The ER stress-mediated apoptosis described above has been demonstrated in colon cancer cell lines; its relevance in vivo remains to be confirmed.

### 5.6. Gallic Acid

Gallic acid is a benzoic acid-type polyphenol with a well-established reputation for antioxidant activity; paradoxically, in cancer cells gallic acid often acts as a pro-oxidant and potent inducer of apoptosis. This dual nature is key to gallic acid’s mechanism. In normal physiological settings, gallic acid can chelate metal ions and scavenge radicals [263]. However, within the high-ROS environment of tumour cells, gallic acid undergoes oxidative conversion to generate semiquinone radicals and hydrogen peroxide, effectively raising intracellular ROS to cytotoxic levels [264]. This surge in ROS causes oxidative DNA damage and dysfunction of mitochondria in cancer cells, overwhelming their redox defences. Consistently, gallic acid has been shown to collapse the mitochondrial membrane potential in a dose-dependent manner and increase lipid peroxidation in cancer cell membranes [265]. The result is activation of the intrinsic apoptotic pathway: gallic acid-treated cancer cells exhibit Bax upregulation, Bcl-2 downregulation, cytochrome c release, and activation of caspase-9 and -3 [266]. In bladder carcinoma T24 cells, for example, gallic acid caused a significant drop in phosphorylated Akt and IκBα, accompanied by increased Bax/Bcl-2 expression, leading to apoptotic cell death [267]. That study linked gallic acid’s effects to simultaneous suppression of the PI3K/Akt/NF-κB pathway, suggesting that gallic acid not only induces direct oxidative injury but also blocks key survival signals. Indeed, gallic acid interferes with multiple cell signalling pathways relevant to cancer progression. It can inhibit constitutive STAT3 and NF-κB activity, reducing transcription of genes involved in proliferation (cyclins, c-Myc), inflammation (COX-2, IL-6), and angiogenesis (VEGF) [268]. Additionally, gallic acid targets receptor tyrosine kinase pathways: in triple-negative breast cancer cells, gallic acid downregulated EGFR expression and attenuated downstream MAPK/ERK signalling, contributing to growth arrest and apoptosis [268,269]. Another facet of gallic acid’s action is the induction of autophagy—some studies report that gallic acid triggers a cytotoxic form of autophagy in cancer cells through AMPK activation and mTOR inhibition, which can synergistically enhance apoptosis [270]. Gallic acid also exhibits antimetastatic properties. It has been noted that it inhibits migration and invasion in melanoma and breast cancer cells by suppressing focal adhesion kinase (FAK) signalling and matrix metalloproteinase expression [271]. In vivo, gallic acid is effective at both reducing tumour growth and potentiating the effects of chemotherapy. For instance, in an A549 lung cancer xenograft mouse model, intraperitoneal 50 mg/kg i.p. gallic acid over 4 weeks significantly reduced tumour volume and when combined with cisplatin led to greater tumour regression than chemotherapy alone [272]. Treated tumour tissues showed increased cleaved caspase-3 and decreased Ki-67 proliferation indices, in line with enhanced apoptosis and cell cycle arrest. Importantly, gallic acid’s pro-oxidant killing of tumour cells does not translate into systemic toxicity when used at moderate doses, owing to normal cells’ higher reserve of antioxidants and lower baseline ROS—a selectivity that is highly advantageous in cancer therapy [273]. In summary, gallic acid combats cancer through a combination of ROS-mediated cytotoxicity and inhibition of survival pathways like Akt, NF-κB, and EGFR/MAPK. This leads to robust apoptosis and can also curb invasion and sensitize tumours to other treatments, marking gallic acid as a uniquely potent polyphenol in the context of cancer.

Although generally considered safe as a dietary component, garlic may interact with anticoagulants due to its antiplatelet properties [274]. Additionally, high antioxidant intake could theoretically attenuate ROS-dependent chemotherapeutic efficacy, as some anticancer drugs rely on oxidative stress mechanisms [275]. Therefore, excessive supplementation during chemotherapy should be approached cautiously.

## 6. Conclusions and Perspectives

Garlic contains polyphenols that exhibit anticancer activity in preclinical models, as demonstrated by extensive in vitro studies. These compounds act through diverse mechanisms: many activate PPAR signalling and the Nrf2 antioxidant response, and a major common mechanism is the induction of ROS-dependent cytotoxicity leading to cancer cell apoptosis. Through these mechanisms, garlic polyphenols exert protective effects by modulating molecular processes involved in cancer initiation and progression. Consistent with this broad activity, regular garlic consumption as part of a healthy diet might help bolster the body’s defences against cancer, owing to its antioxidant, anti-inflammatory, and cell-signalling properties. However, any protective effect of garlic would operate in conjunction with other healthy lifestyle factors rather than as a standalone approach.

Despite these promising mechanisms, the precise anticancer roles of many individual garlic-derived polyphenols remain incompletely understood. Notably, only a few polyphenols in garlic, such as quercetin and apigenin, have progressed beyond cell culture experiments into animal tumour models or preliminary clinical trials, whereas the majority have been evaluated only in vitro. Consequently, robust clinical evidence for their efficacy is still lacking. To our knowledge, no clinical trial to date has conclusively demonstrated anticancer benefits of garlic-derived polyphenols in patients, and current human evidence is limited to epidemiological observations and small-scale intervention studies. Key translational questions remain unanswered—including how to overcome the often poor bioavailability of these compounds to achieve effective systemic concentrations, which dosing strategies or formulations would ensure therapeutic effects in humans, and whether garlic polyphenols can safely synergize with standard chemotherapy regimens to improve treatment outcomes. In our opinion, addressing these uncertainties through focused research such as for example, in advanced animal models followed by well-designed clinical trials, is essential before garlic polyphenols can be reliably integrated into clinical oncology.

Importantly, the molecular targets modulated by garlic polyphenols such as e.g., PI3K/Akt, NF-κB, Nrf2 overlap with those of many existing cancer therapies. This raises the possibility that garlic’s polyphenols could serve as adjunctive agents in cancer treatment or prevention, potentially enhancing the efficacy of conventional treatments. For instance, their influence on key signalling pathways suggests they might be combined with chemotherapy or even newer modalities, like targeted therapies and immunotherapies, to augment anti-tumour effects. However, without robust clinical data, such applications remain speculative, and direct clinical implementation at this stage would be premature. Thus, while preclinical mechanistic data are compelling, further research is needed to determine whether these polyphenol-driven effects can translate into meaningful benefits for patients. Future studies should prioritize specific gaps, such as elucidating how garlic polyphenols affect the tumour microenvironment such as e.g., immune cell infiltration and stromal interactions, exploring synergistic outcomes when garlic-derived compounds are combined with immunotherapies or targeted agents, and identifying biomarkers of garlic polyphenol exposure or efficacy in humans that could guide their clinical use.

To enhance the translational relevance of garlic-derived polyphenols, future research should focus on several key areas. First, well-designed pharmacokinetic and bioavailability studies are needed to determine physiologically achievable concentrations of individual polyphenols and their active metabolites in humans. Second, the development of improved delivery systems, such as nanoformulations, encapsulation, or targeted delivery strategies, may increase stability, tissue accumulation, and therapeutic efficacy. Third, studies using clinically relevant models, including patient-derived tumour organoids and xenografts, could better reflect human tumour biology. Furthermore, combination studies evaluating garlic polyphenols alongside conventional chemotherapeutic agents may help identify synergistic interactions and potential adjuvant applications. The scientific literature particularly emphasizes that the bioavailability and metabolism of polyphenols in the body largely depend on their interactions with the gut microbiota, which has a crucial impact on their biological effects and which should be considered in the design of preclinical and clinical studies. For these reasons, clinical and preclinical studies on polyphenols should include assessments of bioavailability, metabolism in the gastrointestinal tract, and analyses of the gut microbiota and its metabolites—without this, it is difficult to understand which forms of the compound enter the bloodstream and which mechanisms are responsible for the observed effects. Finally, randomized clinical trials investigating standardized garlic preparations or purified polyphenols are essential to confirm their safety, optimal dosing, and clinical efficacy. Addressing these aspects will be crucial for bridging the gap between experimental findings and clinical application. By addressing these issues through rigorous in vivo studies and ultimately clinical trials, we will be better positioned to judge whether the promising anticancer potential of garlic polyphenols can be realized in practice.

## Figures and Tables

**Table 1:** Classification and main dietary sources of flavonoids.

Structural Formula	Types of Flavonoids	Three Example Compounds	Example Sources	References
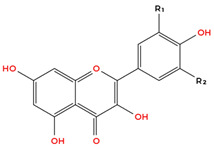	Flavonols	Quercetin, kaempferol, fisetin	Raw/black garlic, red onion, fresh capers	[45,46]
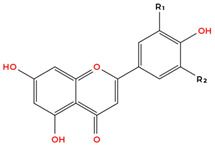	Flavones	Apigenin, acacetin, luteolin	Raw/black garlic, parsley, rosemary	[22,31]
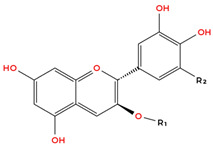	Catechins	Epicatechin, epigallocatechin, epicatechin gallate	Raw garlic, broad beans, green tea	[47,48]
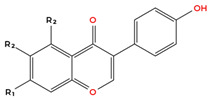	Isoflavones	Daidzein, genistein, glycitein	Raw garlic, soya, broccoli	[49,50]
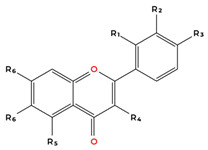	Flavanones	Naringenin, hesperetin, eriodictyol	Raw garlic, orange, lemon	[51,52]
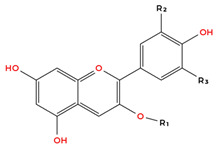	Flavanols	Epigallocatechin gallate, epicatechin, theflavin-3,3′-digallate	Raw/black garlic, green tea, cocoa	[1,53]
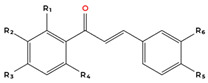	Chalcones	Lonchocarpin, cardamonin, licochalcones	Tomato, *Ginkgo biloba* leaves, liquorice roots	[54,55]

The table summarizes the principal groups of flavonoids and their representative food sources. Not all compounds listed in this classification are present in *Allium sativum* L. Structural formulas were prepared using the online tool MolView https://app.molview.com/.

**Table 3:** Selected anticancer effects of polyphenols detected in garlic on cancerous cell lines in vitro.

Type of Polyphenol	Name of Cell Line	Observed Effect	Reference
Acacetin	MCF-7, 143B, MG63, SJSA, HOS	cell viability↓, apoptosis↑, DNA fragmentation↑, caspase-3, -8, -9↑	[79,80]
Apigenin	SW480, SW620, LS411N	cell viability↓, apoptosis↑, caspase-3↑, PARP cleavage↑, STAT3 phosphorylation↓	[81]
Benzoic acid	HeLa, HUH7, CACO-2, MG63, HT-29, A673, SW48, PC3, HCT-116, HCT-15	cell viability↓, apoptosis↑, ROS↑	[82,83]
Caffeic acid	ME-180, HeLa, HCT-116	apoptosis↑, DNA damage↑, ROS↑	[59,84]
Catechin	HCT-15, HCT-116, HepG2, A549	cell viability↓, apoptosis↑, DNA damage↑, cell cycle arrest↑	[85,86]
Catechol	MCF-7, MDA-MB-231, KP2, H460	DNA damage↑, apoptosis↑, cell cycle arrest↑, colony formation↓	[87,88]
Chlorogenic acid	A549, HT-29	cell viability↓, cell proliferation↓, apoptosis↑, ROS↑	[89,90]
Cinnamic acid	HT-144, CACO-2	cell viability↓, cell cycle arrest↑, apoptosis↑, caspase-9↑, cell number↓, DNA synthesis↓	[91,92]
Coumaric acid isomers	HCT-15, HT-29, U-138MG	cell viability↓, apoptosis↑, DNA fragmentation↑, colony formation↓, ROS↑	[93,94]
Daidzein	MCF-7, Ca9-22, SAS	cell viability↓, apoptosis↑, caspase-3/7↑, ROS↑, Bax↑, Bcl-2↓, migration↓, invasion↓, MMP-2/9↓	[95,96]
Ellagic acid	U87MG, U118, MCF-7, MDA-MB-231	cell viability↓, cell proliferation↓, DNA damage↑, apoptosis↑, cell cycle arrest↑, colony formation↓	[97,98]
Epicatechin	MCF-7, MDA-MB-231, U937, MKN-4	cell viability↓, cell proliferation↓, apoptosis↑, cell cycle arrest↑, ROS↑, DNA fragmentation↑	[99,100]
Epigallocatechin	KKU-M055, A549	cell viability↓ cell cycle arrest↑, apoptosis↑, caspase-8↑, caspase-9↑, caspase-3/7↑, migration↓	[101,102]
Epigallocatechin gallate	H1299, A549, HepG2	cell viability↓, cell proliferation↓, apoptosis↑, colony formation↓, cell migration↓	[103,104]
Ferulic acid	DU-145, MCF-7, PANC-1, MDA-MB-231	cell viability↓, cell cycle arrest↑, cell proliferation↓, apoptosis↑	[58,105]
Gallic acid	A549, HepG2, SMMC-7721	cell proliferation↓, apoptosis↑, DNA fragmentation↑, ROS↑, caspase-3 and -9 expression↑	[106,107]
Hesperidin	HN6, HN15, DU145, PNT1A	cell proliferation↓, cell migration↓, cell viability↓, colony formation↓, ROS↑, LDH release↑, apoptosis↑	[108,109]
Hydroxytyrosol	LS180, Jurkat, HL60, Raw264.7	cell viability↓, apoptosis↑, cell cycle arrest↑	[110,111]
Hyperoside	A431, A432, HS-4, MCF-7, 4T1	cell viability↓, apoptosis↑, cell number↓, cell migration↓, caspase-3 and -9 expression↑	[112,113]
Isoferulic acid	Raji, K562, Jurkat	apoptosis↑, cell cycle arrest↑	[114]
Isoorientin	HT-29, A549, NCI-H23, NCI-H460	cell viability↓, apoptosis↑, caspase-3 and -8 mRNA expression and activities↑, ROS↑, cycle arrest↑	[115,116]
Kaempferol	A375, MDA-MB-231, BT474	cell viability↓, apoptosis↑, cycle arrest↑, colony formation↓, caspase-3 and -9 expression↑	[61,117]
Luteolin	MDA-MB-231, MKN45, BGC823	cell viability↓, apoptosis↑, cycle arrest↑, colony formation↓, caspase-3 and -9 expression↑	[118,119]
Morin	SK-BR-3, SW480	cell viability↓, apoptosis↑, caspase-3 and -7 expression↑, cycle arrest↑, colony formation↓	[120,121]
Myricetin	A549, HT-29	cell viability↓, apoptosis↑, cell migration↓	[62,63]
Naringenin	MDA-MB-231, SKBR3, B16F10, SK-MEL-28	cell viability↓, apoptosis↑, cycle arrest↑, cell migration↓	[122,123]
Naringin	MDA-MB-231, WiDr	cell viability↓, caspase-3 expression↑	[124]
Orientin	T24, HT-29	cell viability↓, apoptosis↑, cycle arrest↑	[125,126]
p-Hydroxybenzoic acid	K562, PC-3, LNCaP, MDA-MB-231, MCF-7, MCF-10A	cell viability↓, apoptosis↑, cycle arrest↑, cell migration↓	[127,128]
Protocatechuic acid	A549, HepG2	cell viability↓	[129,130]
Pyrogallol	HT-29, ASPC-1, A2780, DU145, PC3	cell viability↓, apoptosis↑, cycle arrest↑	[131,132]
Quercetin	DU145, PC3, HepG2, Hep3B, HCT-116	cell viability↓, colony formation↓, ROS↑	[60,133]
Quercitrin	A549, NCI-H358, DLD-1	cell viability↓, apoptosis↑, caspase-3 activity↑	[134,135]
Resveratrol	HK-2, L02, 4T1, HepG2, PC-3	cell viability↓, toxicity↑, apoptosis↑, cycle arrest↑	[136,137]
Rosmarinic acid	A549, MDA-MB-231, U251, U343	cell viability↓, apoptosis↑, cell migration↓, DNA fragmentation↑	[138,139]
Rutin	SAOS2, 786-O	cell viability↓, apoptosis↑	[140,141]
Salicylic acid	B16F10, J774, MCF-7, MCF10A, HCT-116	cell viability↓, apoptosis↑, colony formation↓	[142,143]
Sinapic acid	HT-29, PC-3, LNCaP	cell viability↓, apoptosis↑, caspase-3 expression↑	[144,145]
Syringic acid	Primary gastric cancer, DU-145	cell viability↓, apoptosis↑, ROS↑	[146,147]
Vanillic acid	A549, MCF-7	cell viability↓, apoptosis↑, ROS↑	[148,149]
Vitexin	HCT-116, A549	cell viability↓, toxicity↑, apoptosis↑, caspase-3 expression↑	[150,151]

The table summarises representative in vitro studies describing the modulatory effects of individual garlic-derived polyphenols on cancer cell viability, proliferation, oxidative stress, and apoptosis. Although benzoic acid and cinnamic acid themselves are not phenolic acids, they are included due to their role as metabolic precursors of hydroxybenzoic and hydroxycinnamic acid derivatives, which constitute key subclasses of plant phenolic compounds with recognised anticancer potential. Not all polyphenols listed in Table 2 possess anticancer activity; only those confirmed to do so are included here. ↑, increase; ↓, decrease.

**Table 4:** Major garlic-derived polyphenols and anticancer mechanisms.

Polyphenol	Key Molecular Targets/Pathways	Anticancer Pathways	Evidence	Key Supporting Studies
Quercetin	PI3K/Akt, NF-κB, Nrf2, JAK2/STAT3	caspase-3-dependent apoptosis, autophagy induction, G0/G1 cell cycle arrest, VEGF inhibition (anti-angiogenesis), MMP-9 inhibition (anti-metastasis)	In vitro, In vivo	[60,211]
Caffeic Acid	Nrf2/ARE, PKCδ, MAPK	Nrf2/Activation (antioxidant response), PKCδ-mediated apoptosis, VEGF inhibition (antiangiogenic)	In vitro, In vivo	[212,213]
Ferulic Acid	Cyclins/CDKs, autophagy	cyclin/CDK inhibition (cell cycle arrest); autophagy induction; P-glycoprotein downregulation (chemoresistance reversal)	In vitro, In vivo	[213,214]
p-Coumaric Acid	GRP78/UPR, Bax/Bcl-2, NF-κB	UPR activation (ER stress-induced apoptosis); Bax/Bcl-2 ratio increase (mitochondrial apoptosis); NF-κB inhibition (anti-inflammatory)	In vitro, In vivo	[215,216]
Kaempferol	PI3K/Akt, MMP-2/9, p53	PI3K/Akt pathway inhibition (reduced cell survival); p53-mediated apoptosis; MMP-9 inhibition (antimetastatic); VEGF inhibition (antiangiogenic)	In vitro, In vivo	[217,218]
Gallic Acid	JAK2/STAT3, EGFR/MAPK, ROS	caspase-3/9 activation-dependent apoptosis; JAK2/STAT3 inhibition (chemosensitization); ROS induction (pro-oxidant stress); MMP-9 inhibition (antimetastatic)	In vitro, In vivo	[209,219]

All compounds are present in garlic (*Allium sativum*) or its preparations (including aged/black garlic) and have demonstrated anticancer bioactivity in preclinical studies. Molecular targets refer to the primary proteins or pathways modulated. Anticancer pathways indicate the resultant cellular processes (e.g., apoptosis, cell cycle arrest, anti-invasion) affected by the compound. Abbreviations: ARE—Antioxidant Response Element; Bax/Bcl-2—Bcl-2-associated X protein/B-cell lymphoma 2; CDKs—Cyclin-Dependent Kinases; EGFR—Epidermal Growth Factor Receptor; GRP78/UPR—Glucose-Regulated Protein 78/Unfolded Protein Response; JAK2/STAT3—Janus kinase 2/Signal transducer and activator of transcription 3; MAPK—Mitogen-Activated Protein Kinase; MMP-2/9—Matrix Metalloproteinases 2 and 9; NF-κB—Nuclear Factor kappa-light-chain-enhancer of activated B cells; Nrf2—Nuclear factor erythroid 2–related factor 2; p53—Tumour Protein p53; PI3K/Akt—Phosphoinositide 3-kinase/Protein kinase B; PKCδ—Protein Kinase C delta isoform; ROS—Reactive Oxygen Species.

## Data Availability

No new data were created or analyzed in this study.

## Abbreviations

The following abbreviations are used in this manuscript:

Akt	Protein kinase B
ARE	Antioxidant Response Element
BG	Black garlic
Bax	Bcl-2-associated X protein
Bcl-2	B-cell lymphoma 2
CAT	Catalase
CDKs	Cyclin-dependent kinases
COX-2	Cyclooxygenase-2
DADS	Diallyl disulfide
DAS	Diallyl sulfide
DATS	Diallyl trisulfide
EGFR	Epidermal Growth Factor Receptor
EMT	Epithelial–mesenchymal transition
ER	Endoplasmic reticulum
ERK	Extracellular signal-regulated kinase
FGF	Fibroblast growth factor
GPx	Glutathione peroxidase
GRP78	Glucose-regulated protein 78
HO-1	Heme oxygenase-1
IGF-1R	Insulin-like growth factor 1 receptor
IL	Interleukin
JAK2	Janus kinase 2
JNK	c-Jun N-terminal kinase
LDH	Lactate dehydrogenase
MAPK	Mitogen-activated protein kinase
MMP	Matrix metalloproteinase
mTOR	Mammalian target of rapamycin
NBMA	N-nitroso-N-methylbenzylamine
N.D.	Not detected
NF-κB	Nuclear factor kappa-light-chain-enhancer of activated B cells
Nrf2	Nuclear factor erythroid 2-related factor 2
PARP	Poly(ADP-ribose) polymerase
PI3K	Phosphoinositide 3-kinase
PKC	Protein kinase C
PPAR	Peroxisome proliferator-activated receptor
PPRE	Peroxisome proliferator response element
PTEN	Phosphatase and tensin homolog
ROS	Reactive oxygen species
SAC	S-allylcysteine
SAMC	S-allylmercaptocysteine
SOD	Superoxide dismutase
STAT3	Signal transducer and activator of transcription 3
TNF-α	Tumour necrosis factor alpha
TRAMP	Transgenic adenocarcinoma of the mouse prostate
UPR	Unfolded protein response
VEGF	Vascular endothelial growth factor
